# A bizarre extraoral fistula with intraoral manifestations

**DOI:** 10.1016/S1808-8694(15)31183-6

**Published:** 2015-10-19

**Authors:** Etiene de Andrade Munhoz, Izabel Regina Fischer Rubira Bullen, Eduardo Sant 'Ana, Alberto Consolaro

**Affiliations:** 1 M.S. PhD. Student; 2 Associate Professor - Department of stomatology - Dentistry School of Bauru; 3 Associate Professor - Department of stomatology - Dentistry School of Bauru; 4 Full Professor - Associate Professor - Department of stomatology - Dentistry School of Bauru

**Keywords:** abscess, fistula, hair

## INTRODUCTION

Acute abscess is a common manifestation and needs urgent care. In its advanced stage, it may drain spontaneously through a fistula; it may be extra-oral, depending on the causing tooth, root site, bone thickness and muscle insertions^1^. Spontaneous drainage may leave significant scars. An extra-oral fistula that communicates with the mouth receives constant recontamination^2^.

## CASE PRESENTATION

An 89 year old male patient was referred because of a 30 year old fistula. During exam we noticed skin attachments in the alveolar border.

Periapical and occlusal radiographs were taken and they revealed bone loss. We surgically removed the fistula in order to improve mouth conditions and also to cosmetically improve his face.

At the microscope we noticed hair follicles, sebaceous and sweat glands. The diagnosis was of fistula in a regression stage.

## DISCUSSION

In the literature, authors recommend endodontic treatment as the first option in fistula treatments^2^, ^3^, ^4^, ^5^. However, in some cases, its removal is advocated^1^,^6^.

In this case, since the causing tooth had been removed some thirty years ago, we chose to remove the fistula.

There are not many literature reports on extra-oral fistulas, nor on the presence of skin attachments in the mouth. The single case reported was done by Mitchel6, however this author related it to periodontal disease and a pseudofolliculitis of the beard.

Despite being a bizarre case, treatment was simple and we achieved full intra and extra-oral repair.

## FINAL REMARKS

Understanding the etiopathogenesis of lesions is extremely important for proper diagnosis and treatment, and cases reported with unusual characteristics may help in the diagnosis.
